# From knowledge to attitude: design and initial validation of scales for assessing psychoactive substance consumption among university students

**DOI:** 10.3389/fpubh.2025.1713133

**Published:** 2025-12-16

**Authors:** Riwa Kfoury, Pascale Salameh, Hélène Peyriere

**Affiliations:** 1 PCCEI, Univ Montpellier, INSERM, Univ Antilles, Montpellier, France; 2 Faculty of Medicine, Saint George University of Beirut, Beirut, Lebanon; 3 School of Pharmacy, Lebanese International University, Beirut, Lebanon; 4 Faculty of Pharmacy, Lebanese University, Hadath, Lebanon; 5 Institut National de Sante Publique, Epidemiologie Clinique et Toxicologie - Liban (INSPECT-LB), Beirut, Lebanon; 6 Department of Primary Care and Population Health, University of Nicosia Medical School, Nicosia, Cyprus; 7 School of Medicine, Lebanese American University, Byblos, Lebanon; 8 Department of Medical Pharmacology and Toxicology, Montpellier University, School of Pharmacy, Montpellier, France

**Keywords:** substance use, psychoactive substance consumption, knowledge, attitude, university students, development and validation

## Abstract

**Background:**

The misuse of psychoactive substances among university students has emerged as a pressing public health issue, particularly in Lebanon, where research on this phenomenon is limited. This study aimed to develop and validate scales that assess knowledge and attitudes toward Psychoactive Substance Consumption (PSC), evaluate their psychometric properties, identify factors associated with these scores, and explore the relationship between knowledge, attitudes, and PSC among university students.

**Methods:**

This cross-sectional study surveyed 414 university students from 29 institutions across Lebanon during the 2023–2024 academic year using an anonymous self-administered questionnaire. Participants were recruited via email from three prominent universities with initial Institutional Review Board (IRB) approval, and the sample was expanded through snowballing due to IRB challenges caused by economic and political crises. Exploratory Factor Analysis (EFA) was performed using SPSS to evaluate construct validity, and reliability was assessed by calculating Cronbach's alpha.

**Results:**

EFA identified two factors for the knowledge scale with eigenvalues over 1, explaining 51.4% of the variance. The model demonstrated adequacy, with a Kaiser-Meyer-Olkin (KMO) measure of 0.864, a significant Bartlett's test of sphericity, and a high reliability (Cronbach's alpha = 0.829). Similarly, the attitude scale items converged over two factors, explaining 58.8% of the variance, with a KMO of 0.850, a significant Bartlett's test of sphericity, and good reliability (Cronbach's alpha = 0.826). The study found that greater knowledge was associated with students who had higher grades and those who were aware of the availability of psychoactive substances in nasal inhalation form. However, knowledge showed no significant correlation with attitudes (*r* = −0.027, *p* = 0.583). The multivariate analysis identified several predictors influencing knowledge and attitudes toward PSC, including academic year, financial status changes, presence of mental illness, and family history of substance-related issues.

**Conclusions:**

The scales developed in this study demonstrated strong reliability and validity, positioning them as effective tools for assessing knowledge and attitudes associated with psychoactive substance consumption among university students. The multivariate results underscore the impact of academic, socioeconomic, and mental health factors, suggesting the need for interventions that specifically address these determinants.

## Introduction

Psychoactive substances are defined by the World Health Organization (WHO) as substances that affect the central nervous system, altering behavior, cognition, mood and perception (1). These substances can be legal (e.g., alcohol, tobacco), illegal (e.g., heroin, cocaine), or regulated for use by licensed medical professionals (e.g., antidepressants, anxiolytics, and sleeping medications) which can be used for curative purposes; however, they become misused when they are taken regularly without a physician's prescription (2).

Globally, Psychoactive Substance Consumption (PSC) poses a major public health challenge as it significantly jeopardizes individual health by increasing the risk of chronic diseases as well as mental health disorders (3). Moreover, it also imposes a substantial burden on families and communities, increasing the likelihood of accidents, injuries, and involvement in criminal activities (1).

Despite the well-documented dangers, psychoactive substance consumption persists and, in some contexts, increases. The United Nations Office on Drugs and Crime (UNODC) World Drug Report 2023 stated that over 39.5 million people suffer from substance use disorders, reflecting a 45.0% increase from the previous decade (4).

While PSC can occur at any stage of life, data from the National Center for Drug Abuse Statistics (NCDAS) reveals its highest prevalence among individuals aged 18–25, often referred to as “emerging adults,” with 39.0% reporting use -the highest rate compared to other age demographics (5). Historically, attending university has been considered a protective factor against substance consumption; however, research has shown that university students consume more psychoactive substances than their non-university-attending peers (6). This trend has been exacerbated by the absence of parental supervision, where students are tempted to explore new things (7–9). Several other factors contributing to this vulnerability include stress and anxiety related to academic performance, social life, new responsibilities, separation from family, pressures and concerns about the future, as well as knowledge and perception about substance consumption which also plays a significant role (10–14). Consequently, the excessive consumption of certain substances carries significant risks, including economic and social burdens on families, deteriorating health, disrupted sleep patterns, and a decline in overall quality of life, while also being associated with heightened rates of depression, personality disorders, and even suicide (15–17).

Numerous studies have been conducted evaluating the high rates of PSC among university students worldwide. In the United States, 49.6% of college students consumed alcohol in 2023, while 28.7% admitted to binge drinking (defined as having five or more drinks) (18). Additionally, 18.8% admitted to non-medical use of prescription stimulants (19) and approximately one in four students consumed marijuana or other drugs monthly (20).

In Europe, 73.3% of students consumed alcohol alone or combined with cannabis or other illicit drugs (21), and similar trends were observed in Norway (11), Ethiopia (22), Cameroon (23), and Kenya (24).

In the Middle East, epidemiological studies are relatively scarce but concerning. For example, 28.7% of students at Oujda University in Morocco reported lifetime prevalence of prescription substance consumption (25), while 25.3% of United Arab Emirates (UAE) university students used cognitive enhancers (26). Additionally, sedative consumption was high among Yemeni and Saudi Arabian university students at 12.5% (16).

In Lebanon, psychoactive substance consumption has been a persistent challenge since the 1975 civil war (27, 28). By 2012, nearly one in five university students reported non-medical consumption of prescription medication (29). As the years went by, the situation escalated into a critical issue. By 2014, 20.9% of Lebanese university students reported alcohol consumption, 12.3% used cannabis, and 11.0% took tranquilizers (30). Smoking rates were even more alarming, with over half of university students smoking—far higher than peers in other Arab countries (31). Additional research also confirmed the widespread use of cannabis, opioids, tranquilizers, and stimulants among Lebanese university students, a situation likely exacerbated by ongoing socio-economic challenges and psychological stressors linked to political instability and crises (29–33).

These challenges highlight the need for comprehensive solutions that go beyond prevalence data provided by cross-sectional studies, which cannot explain why these trends persist or how to address them effectively. Studies showed that many students consume these substances due to a lack of awareness regarding their potential negative consequences (34, 35). Additionally, research demonstrated that there is an inverse relationship between knowledge of the risks and perceived harmfulness of these substances and actual usage rates (36, 37). This suggests that the attitudes and desires of university students to consume these substances could be largely driven by ignorance or insufficient understanding of their detrimental effects (10).

The Knowledge, Attitude, And Practice (KAP) theory is widely utilized to examine the impact of individual knowledge and beliefs on health behavior modifications. It highlights the complex relationship between knowledge and behavior, emphasizing that changing attitudes is essential for facilitating modifications in health practices (38, 39). To date, research on the development and validation of KAP scales regarding psychoactive substance consumption in Lebanon remains limited. While some studies have explored waterpipe smoking—a culturally significant practice that raises considerable public health concerns (40)—other investigations have focused on the validating KAP instruments in specific context, such as tranquilizer misuse (41), cannabis consumption (35) along with recent research KAP studies concerning opioids use (42). However, existing tools either primarily target specific substances or focus on risk assessment and severity of consumption rather than exploring knowledge and attitudes across a broader spectrum of psychoactive substances, leaving a critical gap in understanding how educational deficits and cultural perceptions shape university students' behaviors toward psychoactive substances in Lebanon. This limitation underscores the need for developing validated scales that systematically measure knowledge and attitudes across a broader spectrum of substances and would fill this gap by uncovering misconceptions and cultural norms that drive substance consumption behaviors among this population. This approach aligns with Lebanon's Inter-ministerial Substance Use Response Strategy, emphasizing the importance of evidence-based approaches to strengthen prevention efforts (43).

The primary objective of this study is to develop comprehensive scales that effectively assess the knowledge and attitudes of university students regarding psychoactive substance consumption (PSC) and to validate these scales by examining their psychometric properties; a secondary objective is to evaluate factors associated with these scores, focusing primarily on socio-demographic characteristics, economic status, and mental health.

## Materials and methods

### Study design

This cross-sectional study was conducted to validate knowledge and attitude scales regarding various psychoactive substances, while also assessing their psychometric properties among Lebanese university students. A total of 438 undergraduate university students participated in the study while actively enrolled and attending universities across various regions of the country. Of these respondents, only 414 met the inclusion criteria, specifically those aged between 18 and 25 years. A self-administered Web survey was distributed during the academic year 2023–2024 to university students all over Lebanon. The sample was distributed as follows: 90.2% of participants attended eight major Lebanese universities (one public and seven private institutions with mixed tuition fees, reflecting socioeconomic diversity), while the remaining 9.8% were enrolled in smaller universities. Exclusion criteria included students who did not consent, those who did not complete the survey, those who are below 18 and above 25 years of age, and those who are not residents of Lebanon.

### Sample size calculation

The sample size was calculated using Epi-info™ 7 (Center for Disease Control, Atlanta, GA, USA), considering a confidence interval of 95% and a margin of error of 5% (44). By using 50% of the total estimated population of 243,953 enrolled university students according to the statistical bulletin-Center for Educational Research and Development (CRDP) in Lebanon, the minimal needed sample size was 384 students.

Before starting the main data collection, we conducted a pilot study with 20 students from one university. These students were recruited through an email that provided a short description of the study and a link to the questionnaire. Students were explicitly instructed not to participate again if they received subsequent invitations. The purpose of the pilot was to assess how long it took to complete the questionnaire and to check if the questions were clear and easy to understand. The participants found the questionnaire's format, design, and ease of use satisfactory and didn't suggest any major changes. The data collected from the pilot study was not included in the final database.

### Questionnaire and variables

The questionnaire used in this study was available in English and was composed of a brief introduction and three main sections. The introduction included the study's objective, statement of privacy, and consent request from each participant before proceeding to the next sections.

Section I comprised the socio-demographic and background characteristics (such as age, sex, marital status, place of residence, level of education, employment status, etc.). Additionally, it incorporated the financial wellbeing scale developed by the Consumer Financial Protection Bureau (CFPB). This scale is a validated 10-item instrument using a five-point Likert scale ranging from 0 to 100, where higher scores reflect greater financial wellbeing. This standardized measure is essential for identifying students at risk of financial distress (45, 46). On another note, students were asked to classify their financial situation by comparing their self-reported status in 2019 (before the economic, and political crisis as well as the COVID-19 pandemic) with their status in 2024 (the time of data collection). To analyze the changes in financial status over this period, a variable of difference named “Diff” was calculated as the difference between financial status in 2024 and 2019. The variable provided insights into the financial shifts experienced by the students, with a value of zero, indicating no change in financial status over the 5-year span.

Section II covered the student's health characteristics, including the Generalized Anxiety Disorder-7 (GAD-7) and Patient Health Questionnaire-9 (PHQ-9), both validated in Lebanon with demonstrated reliability and cultural appropriateness (47).

- GAD-7 is a seven-item scale assessing the severity of GAD. Each item in this scale is scored from 0 (not at all) to 3 (nearly every day) resulting in a total score between 0 and 21. Higher scores indicate a more severe anxiety (48).
- PHQ-9 is a nine-item scale that measures the severity of depressive symptoms. Scores range from 0 to 27 with high scores indicating a more severe depression (49).

Section III focused on the Knowledge, Attitudes, and Practices of university students toward the consumption of Psychoactive substances.

- The knowledge and Attitudes constructs were designed to consist of items that measure the understanding of various psychoactive substances and their effects. Some relevant questions were sourced from existing literature (50–52), while others were developed by the authors to incorporate a more comprehensive overview of students' understanding of various substances. The face and content validity measures were assessed by the authors, who are a pharmacist with public health specialty, a researcher with long experience in drug abuse research, and a pharmacist and epidemiologist with long experience on epidemiologic research. While our team assessed content validity, future research could benefit from external expert input to further strengthen the measures. A consensus was reached regarding the relevance, importance, and comprehensiveness of the items included in the questionnaire before piloting. Students answered on a 5-point Likert scale; the correct answer scored the highest point (4) and the wrong answer scored zero. The knowledge scale consisted of 11 questions (score ranges between 0 and 44) and the attitude score included 10 questions (score ranges between 0 and 40).
- The Practice questions were adopted from the validated Alcohol, Smoking and Substance Involvement Screening Test (ASSIST) tool which was developed by the World Health Organization (WHO) to assess an individual's substance consumption patterns and associated problems. This tool consists of eight core questions evaluating the substance consumption, related problems and symptoms of dependance (53). The ASSIST tool has been validated in Lebanon, ensuring its relevance and reliability in this context (42).

### Sampling process

The initial participants in the questionnaire were students from the universities that granted Institutional Review Board (IRB) approval, they were approached via an email containing a brief description of the study along with a link to the questionnaire. These participants then referred additional students to participate, thereby expanding our sample size. This snowballing approach was particularly effective given the challenges faced during the data collection period, as some universities provided IRB approval for our research, while others did not. The lack of IRB approval in certain institutions was largely due to the country's ongoing economic and political crisis, which resulted in frequent closures of university administration offices. Despite these obstacles, we successfully gathered data from a diverse group of students, ensuring a comprehensive understanding of their perspectives.

### Statistical analysis

Statistical analysis was done using Statistical Package for Social Sciences (SPSS), version 27. Descriptive statistics were calculated using mean and Standard Deviation (SD) for normally distributed continuous variables. To check the normality of the knowledge and attitude scores, skewness and kurtosis were conducted with accepted values ranging from −2 to +2. These conditions assert normality assumptions in samples of more than 300 (54, 55).

Associations between knowledge and attitude scores with dichotomous variables such as gender, marital status, exercise, and work status were compared using the Pearson chi-square test, and when the expected values within cells were < 5, the Fisher exact test was used. Furthermore, bivariate correlations were analyzed using the Student's *t*-test. A *p-value* < 0.05 was considered significant for all the statistical analyses carried out.

Exploratory factor analysis (EFA) of the knowledge and attitude scales was conducted. The range of factors/components was defined with the help of their comparison by parallel analysis. The suitability of data for factor analysis was determined through the Kaiser-Meyer-Olkin (KMO) test and Bartlett's test of sphericity. Factors with eigenvalues >1 were retained and for each test item, the minimum cutoff value for factor loading was determined at 0.4 (56). Cronbach's alpha coefficients were computed to analyze the reliability of the knowledge and attitude scales and their subsequent subscales. A Cronbach's alpha above 0.7 suggests high internal consistency (57, 58).

## Results

### Description of sociodemographic characteristics

A total of 438 university students participated in this study, of whom 414 (94.5%) were between 18 and 25 years of age and these were included in our study. The mean age was 21.1 ± 1.92 years with 57.7% female. The marital status of the students was predominantly single (95.9%) with a small fraction being married (3.4%) and only a minority reported being widowed or divorced (0.7%). Of the participants included, the large majority of the respondents studied in Beirut (63.0%), followed by Mount Lebanon (27.8%) and a significant proportion of students resided off-campus with their families (78.7%). Last, most of the respondents (65.2%) were students only; the others were students working part-time (19.6%), full-time (12.3%), or trainees (2.9%). These socio-demographic, economic, and other background characteristics, including gender, marital status, residence, student status, and financial wellbeing score are summarized in Table 1 below.

**Table 1:** Sociodemographic and background characteristics of Lebanese university students participating in the study (*n* = 414).

**Variable**	***n* (%)**
**Gender**	
Male	175 (42.3)
Female	239 (57.7)
**Marital status**	
Married	14 (3.4)
Single	397 (95.9)
Widowed/divorced	3 (0.7)
**Current campus location**	
Beirut	261 (63.0)
Mount Lebanon	115 (27.8)
North	15 (3.6)
South	7 (1.7)
Nabatiyeh	2 (0.5)
Bekaa	14 (3.4)
**Where do you live?**	
On-campus alone	11 (2.7)
On campus with roommate	39 (9.2)
Off-campus alone	37 (8.5)
Off-campus with roommate	4 (1.0)
Off-campus with family	347 (78.7)
**Work status**	
Student only	270 (65.2)
Employed part-time and student	81 (19.6)
Employed full-time and student	51 (12.3)
Trainee/Intern and student	12 (2.9)
**Grades**	
Good/very good (A/B)	289 (69.8)
Passing (C)	108 (26.1)
Failing (D/F)	17 (4.1)
**Major of study**	
Health sciences major	221 (53.4)
All other majors	193 (46.6)
**Current academic year**	
First year	82 (19.8)
Second year	104 (25.1)
Third year or more	228 (55.1)
**Parents marital status**	
Parents living together	348 (84.1)
Parents not living together (D/W/S)	66 (15.9)
**Mother's education level**	
Postgraduate	65 (15.7)
Graduate	175 (42.3)
Undergraduate	174 (42.0)
**Father's education level**	
Postgraduate	66 (15.9)
Graduate	154 (37.2)
Undergraduate	194 (46.9)
**Difference in financial status 2024–2019 “Diff”**	
Diff = 0 (no change)	115 (27.8)
Diff < 0 (negative)	268 (64.7)
Diff > 0 (positive)	31 (7.5)
**CFPB score ranges**	
Very low (0–29)	2 (0.5)
Low (30–37)	16 (3.9)
Medium low (38–49)	112 (27.1)
Medium high (50–57)	184 (44.4)
High (58–67)	84 (20.3)
Very high (68–100)	16 (3.9)

### Financial wellbeing

Table 1 shows that a significant portion of students fall within the Medium-High category on the CFPB scale indicating that 44.4% of students have a relatively stable financial situation, are able to cover emergencies and have some level of savings.

Regarding the changes in financial status, 27.8% of the students had no change in financial shifts between 2024 and 2019. In contrast, the majority representing 64.7%, had a negative Diff value, suggesting a regression in their financial status during these past 5 years.

### Mental health indicators

The GAD-7 score range and its interpretation reveal a notable prevalence of anxiety symptoms among participants. As detailed in Table 2, the reported mean score of GAD-7 was 10.07 ± 6.187, suggesting that, on average, students fall into moderate levels of anxiety. Also, the distribution of score ranges highlighted that 33.6% of the students are affected by mild anxiety, while 26.6% experience severe anxiety.

**Table 2:** Description of health characteristics and assessment scores of Lebanese university students participating in the study (*n* = 414).

**Variable**	***n* (%)**
**Exercise**	
No	177 (42.8)
Yes	237 (57.2)
**Mental health issues**	
No	399 (96.4)
Yes	15 (3.6)
**Family member suffering from the following illness**	
Mental health illness	29 (7.0)
Heavy smoking	98 (23.7)
Alcoholism	7 (1.7)
Drug abuse	6 (1.4)
Family member (parent/sibling) has more than one of the above	14 (3.4)
**GAD score range**	
None to minimal anxiety	79 (19.1)
Mild anxiety	139 (33.6)
Moderate anxiety	86 (20.8)
Severe anxiety	110 (26.6)
**PHQ-9 score range**	
Minimal depression	79 (19.1)
Mild depression	141 (34.1)
Moderate depression	81 (19.6)
Moderately severe depression	60 (14.5)
Severe depression	53 (12.8)
**Knowledge score**	
Mean ± SD	32.77 ± 6.319
Median (25th percentile; 75th percentile)	33.00 (29; 37)
Min	0
Max	44
**Attitude score**	
Mean ± SD	21.92 ± 7.622.
Median (25th percentile; 75th percentile)	23.00 (19; 27)
Min	0
Max	40

Additionally, the PHQ-9 score assessing the presence and severity of depressive symptoms, indicated a mean score of 10.82 ± 6.981 which according to the established scoring guidelines, places students in the moderate depression category. Moreover, the distribution of the score range denoted that a substantial portion of participants (~54%) experienced mild to moderate depressive symptoms. The comprehensive range of health characteristics and assessment scores is presented in Table 2, offering a complete overview of the mental health profile of Lebanese university students participating in the study.

### Exploratory factor analysis (EFA)

#### Knowledge toward psychoactive substance consumption (PSC)

**Figure 1:**
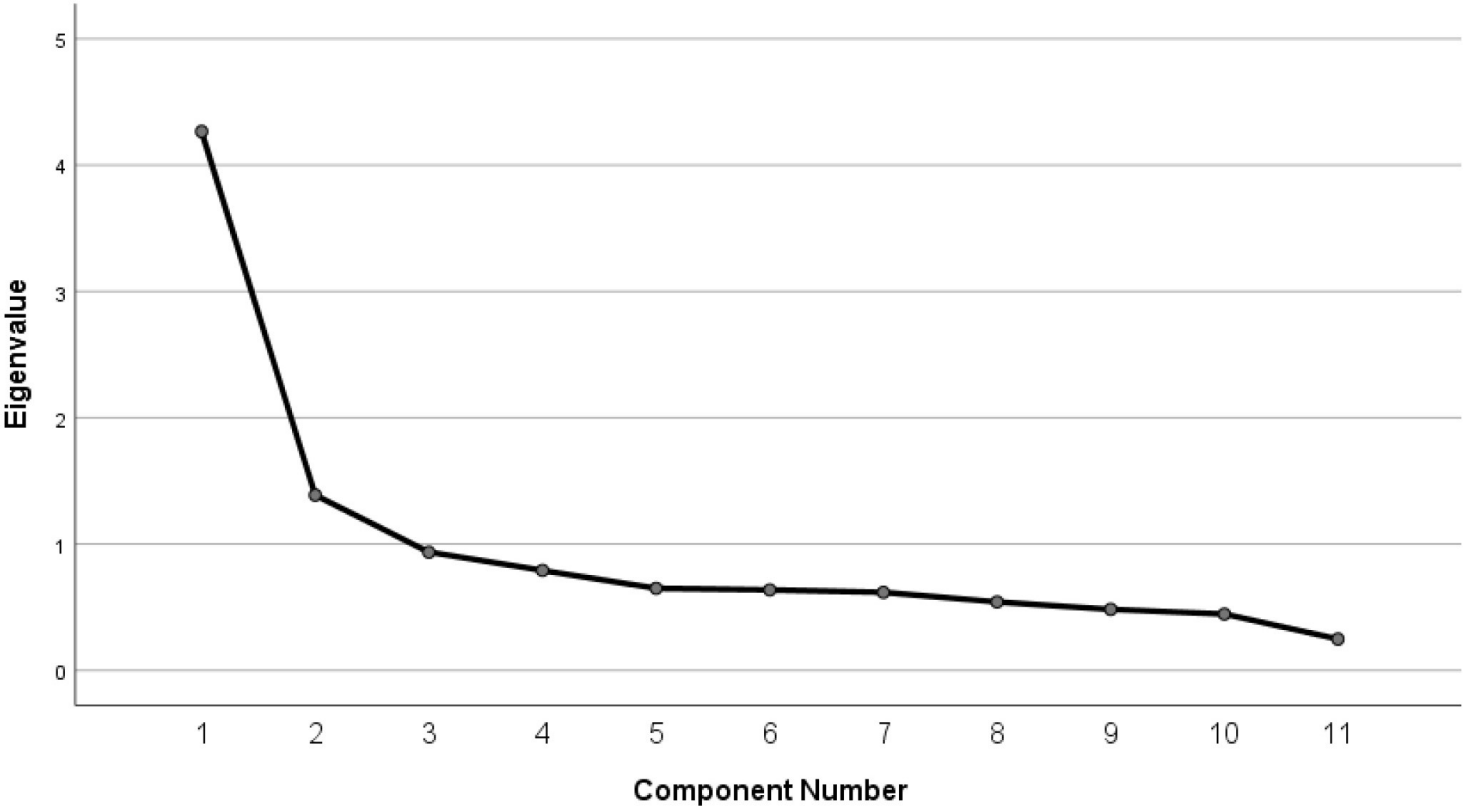
Scree plot depicting factor retention in EFA of the knowledge scale toward PSC. The plot displays the eigenvalues associated with each factor extracted during EFA. The inflection point (“elbow”) suggests that two factors should be retained, as both have eigenvalues >1.

Factor analysis showed that the knowledge construct explained a total of 51.4% of the variance indicating that a substantial portion of the variability in the data can be accounted for and that items are effectively capturing the underlying knowledge construct. As illustrated in Figure 1, the scree plot displays the proportion of variance explained by each factor, with the elbow point indicating that the first two factors account for the majority of variance. This pattern supports the adoption of a two-factor model in EFA; factor 1 corresponds to perception and consequences of PSC and factor 2 relates to psychosocial influences of PSC. The rotated component matrix, presented in Table 3, details the factor loadings for each item, illustrating how individual scale items contribute to the two identified factors. The total KMO was 0.864 with a significant Bartlett's test of sphericity (χ^2^ = 1,403.680; *p* < 0.001) indicating that the underlying structure of data is well-suited for factor extraction. The internal consistency was assessed using Cronbach's alpha, which yielded a value of 0.829 indicating a strong level of internal consistency among the scale items.

**Table 3:** Rotated component matrix^a^ for exploratory factor analysis of the knowledge scale.

**Knowledge questions**	**Component**	
	**Factor 1 perception & consequences of PSC**	**Factor 2 psychosocial influences on PSC**
1. Psychotropic substances may cause changes in mood, awareness, thoughts, feelings, or behavior	0.850	
2. Psychotropic substance consumption affects how the brain works	0.825	
3. When people engage in substance consumption, they get a lift and feel of instant happiness and pleasure	0.685	
4. The rate of drug abuse has increased over the years	0.665	
5. Peer pressure influences substance abuse	0.649	
6. Health problems can result from taking psychotropic substances	0.636	
7. Students who abuse drugs have low self-esteem		0.731
8. Family background is a leading cause of substance abuse		0.660
9. High consumption of marijuana decreases sexual hormones		0.610
10. Psychotropic abuse makes you more vulnerable to HIV & unwanted pregnancy		0.608
11. Women have low tolerance to alcohol than men		0.584

Extraction method: principal component analysis.

Rotation method: varimax with Kaiser normalization.

^a^Rotation converged in three iterations.

#### Attitude toward psychoactive substance consumption (PSC)

**Figure 2:**
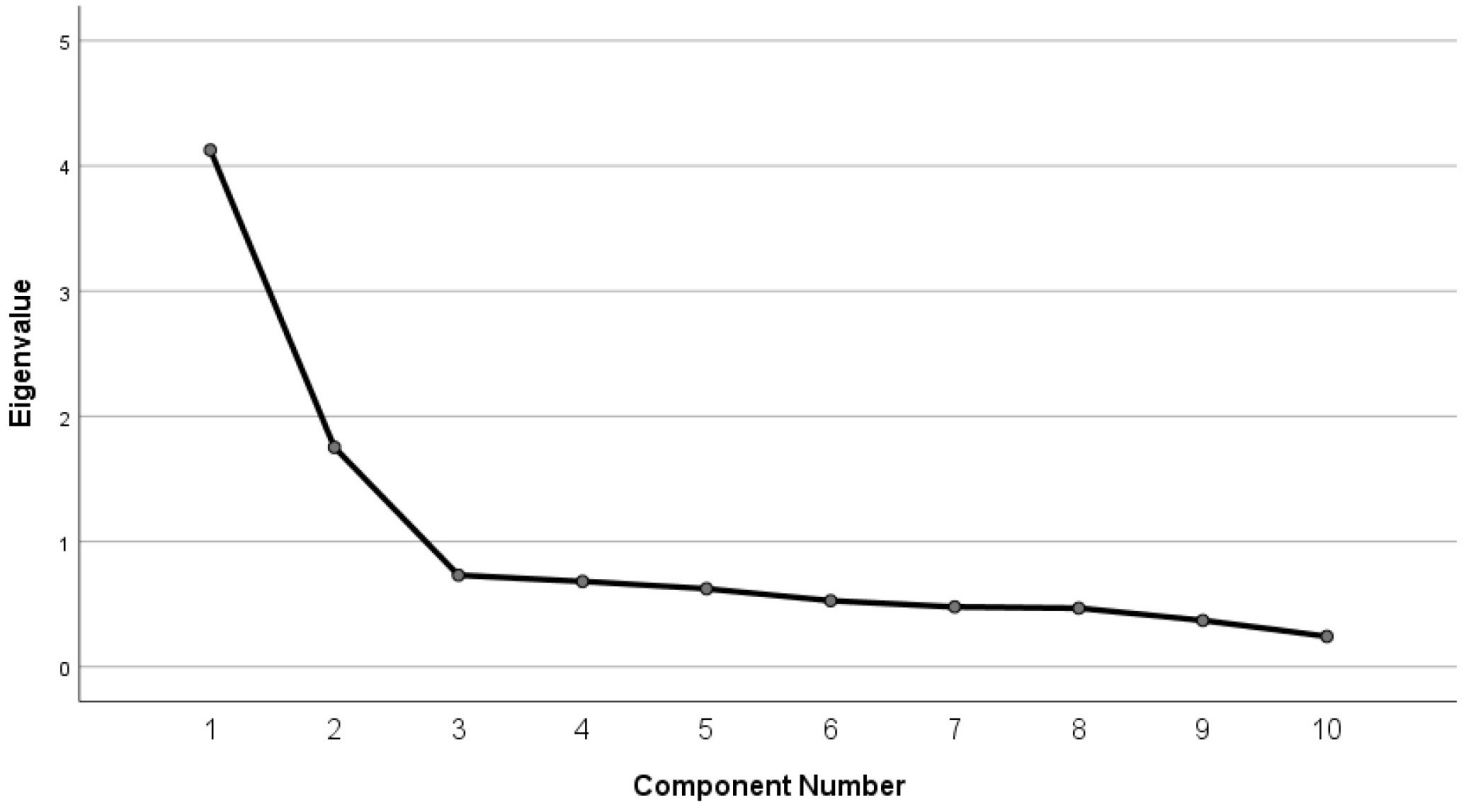
Scree plot depicting factor retention in EFA of the attitude scale toward PSC. The plot shows the eigenvalues for each extracted factor. The “elbow” in the plot indicates that two factors have eigenvalues >1, supporting their retention.

EFA analysis of the attitude scale accounted for a total variance of 58.8%, indicating that the scale items collectively explain a substantial proportion of the variability in students' attitudes toward PSC. As illustrated in Figure 2, the scree plot supports the selection of a two-factor model, with the elbow point indicating that the first two factors account for the majority of variance. This two-factor structure is further detailed in Table 4, which presents the rotated component matrix and demonstrates that factor 1 corresponds to behavioral intent toward PSC, while factor 2 relates to social norms regarding PSC. The KMO measure was 0.850, and Bartlett's test of sphericity was highly significant (χ^2^ = 1,529.402; *p* < 0.001), confirming that the data structure is well-suited for factor extraction. Cronbach's Alpha coefficient yielded a value of 0.826, reflecting a good level of reliability for the scale and supporting the differentiation of attitudes across the identified factors.

**Table 4:** Rotated component matrix^a^ for the exploratory factor analysis of the attitude scale.

**Attitude questions**	**Component**	
	**Factor 1 behavioral intent toward PSC**	**Factor 2 social norms regarding PSC**
1. Do you feel comfortable taking psychotropic substances without physician's prescription?	0.839	
2. Do you intend to use any substance in the next 6 months?	0.837	
3. Taking psychotropic medication without prescription from time to time is not harmful	0.757	
4. The benefit of taking these substances outweigh their risks	0.742	
5. Do you feel pressured to take any kind of substance by your friends?	0.725	
6. Are these substances readily available to you?	0.687	
7. Do you think you have all the information you need about psychotropic substances?	0.561	
8. Substance abuse is a crime and should be punishable by law?		0.837
9. Do you think that students who abuse these substances are often violent?		0.812
10. Do you believe that most students who abuse these substances come from poor backgrounds?		0.635

Extraction method: principal component analysis.

Rotation method: varimax with Kaiser normalization.

^a^Rotation converged in three iterations.

Using the revised Bloom's taxonomy, a cut off score of 75% was applied to categorize students into two groups: poor/good knowledge and negative/positive attitudes (59). The results indicated that a concerning proportion of 72.5% of students demonstrated poor knowledge and 71.1% exhibited negative attitudes toward psychoactive substance consumption.

### Bivariate analysis

A bivariate analysis was conducted in this study to explore the relationships between the categorical and continuous variables related to knowledge and attitudes toward psychoactive substance consumption. As shown in Table 5, the statistical analysis identified only two variables that were significantly associated with the knowledge score.

**Table 5:** Summary of key bivariate analysis of categorical variables with the knowledge and attitude scores.

**Students' characteristics**		**Knowledge [*****n*** **(%)]**		***p*-Value**
		**Poor knowledge**	**Good knowledge**	
Students' grades	Good grade (A or B)	204 (70.6)	85 (29.4)	0.030
	Passing grade (C)	79 (73.1)	29 (26.9)	
	Failing grade (D or F)	17 (100.0)	0 (0.0)	
Students aware of nasal inhalation (e.g., snorting, nasal spray, etc.)	No	51 (83.6)	10 (16.4)	0.035
	Yes	249 (70.5)	104 (29.5)	

Notably, there was an association between knowledge levels across different grade categories as indicated by a chi-square test value of χ^2^ = 6.995, with a *p-*value of 0.03. These findings underscore the importance of academic performance in relation to knowledge of psychoactive substances, suggesting that as academic performance improves, so does knowledge in this area. Another association was observed concerning the students' awareness of the availability of psychoactive substances in nasal inhalation form (e.g., snorting), showing a chi-square test value of χ^2^ = 4.452 and a *p*-value of 0.035. However, no associations were found between the attitude score and any of the variables included in the questionnaire. A comprehensive overview of all bivariate relationships is provided in Supplementary Table S5, which is available in the Supplementary material at the end, facilitating a clear understanding of the factors influencing knowledge and attitudes toward psychoactive substance consumption among the study participants.

Correlation analysis between the two scores of knowledge and attitude was conducted. Results showed that there is almost a negligeable negative correlation between knowledge and attitudes toward psychoactive substance consumption among Lebanese university students with a Spearman coefficient of −0.027 and a *p-*value of 0.583. These findings imply that knowledge about PSC does not necessarily influence students' attitudes toward their use.

### Multivariate analysis

The findings from the multiple linear regression analysis identified several significant predictors of students' knowledge and attitude scores, as detailed in Table 6. For knowledge score, a positive correlation was observed with the student's academic year (beta coefficient = 1.822; *p-*value = 0.008); the difference in financial status between 2024 and 2019 or “Diff” (beta coefficient = 0.688; *p-*value = 0.034); the presence of mental illness (beta coefficient = 5.935; *p-*value = 0.043) and the impact of anxiety problems on students' ability to work, take care of things at home or get along with other people (beta coefficient = 2.483; *p-*value = 0.002). In contrast, suffering from asthma or allergies was negatively associated with the knowledge score (beta coefficient = −5.243; *p-*value = 0.028); as well as if the students had a family member (parent, brothers or sisters) who had more than one of the following issues: mental illness, alcoholism, substance abuse or heavy smoking as defined by consuming 20 or more cigarettes daily (beta coefficient = −9.339; *p-*value = 0.004).

**Table 6:** Multiple linear regression analysis of knowledge and attitude scores.

**Independent variables**	**Unstandardized coefficients**		***p*-Value**	**95% CI**
	β	**Std. error**		
**Knowledge score**				
Academic year	1.822	0.684	0.008	[0.472; 3.171]
“Diff”	0.685	0.321	0.034	[0.051; 1.319]
Mental illness	5.935	2.908	0.043	[0.198; 11.671]
Having asthma or allergy	−5.243	2.365	0.028	[−9.907; −0.578]
Family member (parent or sibling) has more than 1 disease (mental illness, alcoholic, heavy smoker, drug abuse)	−9.339	3.222	0.004	[−15.694; −2.984]
How difficult have anxiety problems made it for students to work, take care of things at home or get along with other people	2.483	0.805	0.002	[0.896; 4.069]
**Attitude score**				
Academic year	1.664	0.814	0.042	[0.058; 3.269]
Location of university	−1.166	0.421	0.006	[−1.996; −0.336]

For the attitude score, there was a positive correlation with the students' academic year (beta coefficient = 1.664; *p*-value = 0.042); and a negative one with the location of the university (beta coefficient = −1.166; *p-*value = 0.006). Table 6 offers a complete summary of these multivariate regression results, highlighting the main factors that influence students' knowledge and attitudes toward PSC.

## Discussion

This study evaluated the knowledge and attitude toward psychoactive substance consumption among Lebanese university students. It also validated a questionnaire specifically designed to address these factors. To our knowledge, this is the first study focused on designing and validating scales to understand the perceptions of psychoactive substances among university students in Lebanon. While previous research has provided valuable insights into specific substances (35, 40–42), our study fills a crucial gap by offering a comprehensive assessment of knowledge and attitudes toward a broader range of psychoactive substances among this vulnerable population.

The participant analysis revealed that the majority of respondents had an average age of 21.1 ± 1.9 and were predominantly female and single (Table 1). The high percentage of students residing off-campus with families may suggest a protective factor against substance consumption, as familial support could mitigate the risks associated with independent living, such as behavioral problems and substance consumption (60).

As previously mentioned, Lebanon has encountered a multitude of challenges since 2019, resulting in a significant rise in psychological distress among its population. The combination of economic and financial collapse that began in late 2019, COVID-19 pandemic, the devastating Beirut explosion on August 4, 2020—the world's most powerful non-nuclear explosion of the 21st century—and the recent war in 2024 has significantly aggravated this situation (61–64). This distress is particularly pronounced among university students, who are further burdened by academic pressures, socio-economic stressors, and concerns about their future (10–12).

While financial pressure is a notable factor contributing to this stress and understanding its implication can enhance our insight into how financial wellbeing affects health outcomes and behaviors related to substance consumption and misuse (46, 65), many students reported a relatively stable financial situation as indicated by the CFPB financial wellbeing scale, with 44.4% of students falling within the Medium-High category. This stability could positively influence their overall wellbeing and ability to manage academic demands. However, it is concerning that a substantial majority experienced a regression in their financial situation over the past 5 years. This decline, likely reflecting the political crisis, economic challenges, the pandemic, the wars and other downturns that the country has suffered from in the past 5 years, may contribute to increased stress and anxiety, potentially leading to higher vulnerability to substance consumption as a coping mechanism.

Our research findings align with the existing literature, indicating an alarming prevalence of anxiety and depressive symptoms among participants, with mean GAD-7 scores indicating moderate anxiety levels and PHQ-9 scores suggesting moderate depression (Table 2). This finding supports previous studies that have established a connection between mental health challenges and increased substance consumption. Notably, a significant portion of students (53.7%) is experiencing mild to moderate depressive symptoms, which may place them at a heightened risk for using psychoactive substances as means of self-medication (3). Therefore, it is evident that mental health issues are prevalent among university students, underscoring the vulnerability of this demographic and the need for mental health support for these emerging adults.

### Reliability and validity of knowledge and attitude scales

The Exploratory Factor Analysis (EFA) provided valuable information on the psychometric properties of the knowledge and attitude scales toward substance consumption. The identification of two distinct factors for both scales suggests that students distinguish between the consequences of substance consumption and the social expectations surrounding it (Tables 3 extbfand 4). The concerning statistics indicated that 72.5% of students demonstrated poor knowledge and 71.1% exhibited negative attitudes toward psychoactive substances. These findings highlight an urgent need for educational programs that not only provide information but also foster critical thinking and understanding of substance consumption and misuse.

The knowledge construct explained 51.4% of the variance in the score indicating that the items effectively capture the underlying scale of knowledge about psychoactive substances. The KMO value of 0.864, with a significant Bartlett's test of sphericity, falls into the “meritorious” sampling adequacy according to KMO interpretation guidelines (66). This indicates that the intercorrelation among the proposed variables is sufficient to allow factor extraction, suggesting a well-structured scale for measuring knowledge. Similarly, the attitude scale demonstrated a total variance explanation of 58.8% with a KMO of 0.850 indicating a good sampling adequacy for factor analysis.

Moreover, the Cronbach alpha values of 0.829 for knowledge and 0.826 for attitude suggest strong internal consistency among these scales' items, affirming their reliability in measuring the knowledge and attitude toward psychoactive substance consumption.

The Bivariate analysis showed significant associations between knowledge levels and awareness regarding the availability of psychoactive substances in inhalation forms, as well as with students' academic performance (Table 5). Among students who recognized that psychoactive substances can be inhaled, 70.5% demonstrated poor knowledge, while 29.5% exhibited good knowledge. In contrast, among those unaware of these inhalation forms, a much higher percentage, 83.6%, had poor knowledge, with only 16.4% showing good knowledge. This data indicates that, while awareness of inhalation forms is linked to better knowledge, a significant number of students still lack a comprehensive understanding of these substances.

Results also show a potential correlation between academic achievement and knowledge about consumption of psychoactive substances. Students with higher academic grades exhibited a greater understanding of psychoactive substances. Specifically, 29.4% of students with very good grades demonstrated good knowledge, compared to 26.9% of those with passing grades. Predominantly, all students with failing grades displayed poor knowledge. These findings point an aspect for further exploration in educational strategies potentially leading to the development and integration of structured educational programs into the university curriculum to address the types of psychoactive substances, their effects, risks and legal implications. Additionally, implementing workshops, seminars or awareness campaigns could facilitate discussions about substance consumption.

Notably, no associations were found between attitude scores and any questionnaire variables. This may denote a potential gap in understanding how attitudes are formed or are influenced by other factors that were not measured in our study.

The correlation between knowledge and attitude scales revealed a negligible negative correlation between knowledge and attitudes toward psychoactive substance consumption (Spearman coefficient = −0.027; *p* = 0.583). This finding suggests that increased knowledge does not necessarily correlate into more positive attitudes toward substance consumption among Lebanese university students, which may reflect deeper cultural or contextual factors influencing their perceptions such as cognitive dissonance, emotional vulnerability or peer pressure (6, 67, 68).

The multivariate analysis identified several predictors influencing knowledge and attitudes toward psychoactive substance consumption, including academic year, financial status changes, the presence of mental illness, and family history of multiple substance-related issues (Table 6). Results indicated that as students progress through their academic years, their knowledge and attitude scores tend to increase significantly, likely reflecting the accumulation of learning and experience. Additionally, improvement in their financial status over time is associated with higher knowledge scores as well. Unexpectedly, a negative association was found between having asthma or allergies and knowledge regarding psychotropic substances. Although this finding lacks a clear theoretical explanation, it may reflect differing health priorities or engagement with health information among students with chronic conditions. However, this warrants further investigation in future studies. Results also showed that students reporting mental health challenges may have developed coping mechanisms that enhance their knowledge acquisition or retention. Furthermore, students from families with multiple health issues such as mental illness, substance abuse, alcoholism or heavy smoking tend to lack critical knowledge that could help them avoid these pitfalls. Regarding anxiety, despite the difficulties it creates for students in managing work, household responsibilities, and interpersonal relationships; there was a positive correlation with knowledge score suggesting that anxiety may motivate some students to seek additional resources or support, thereby enhancing their learning outcomes. The multivariate analysis for the attitude scale retained only two significant predictors, academic year and location of university. Despite these significant associations, the model showed limited explanatory power overall, reflecting the complex and multifaceted nature of attitude formation. Attitudes are influenced by a broader range of factors beyond those measured in this study, likely including cultural norms, religious beliefs, and other social and psychological determinants. This limited variance explained underscores the importance of incorporating such broader influences in future research to more fully understand attitude formation in this context.

### Limitation

Based on our research, this article represents the first study dedicated to the development and validation of scales that assess the knowledge and attitude of Lebanese university students regarding psychoactive substance consumption. However, there are several limitations to consider. The cross-sectional nature of the data restricts our ability to establish causal relationships, as the data captures information at a single point in time. Additionally, the use of a self-administered questionnaires may introduce a risk of information bias as respondents may misinterpret questions and provide inaccurate answers; although this bias should be minimal due to the pilot testing carried out at the beginning of survey dissemination which helped rephrase some questions that were thought to be misunderstood.

Although our sample was drawn from universities across Lebanon and included an adequate number of participants (414 students) for statistical analysis, the recruitment method may have introduced selection bias. Snowball sampling is effective for reaching hard-to-access students, especially during the challenging period when campuses were closed or difficult to reach. Nonetheless, this method likely increased the participation of students more concerned about substance consumption or those within certain social networks, which may not fully represent all Lebanese university students. Another concern could be social desirability bias which could lead students to provide answers they believe are more acceptable rather than their true behaviors, particularly regarding sensitive topics like substance consumption.

Another methodological limitation of this study is that only Exploratory Factor Analysis (EFA) was conducted to assess the underlying structure of the knowledge and attitude scales. EFA was deemed appropriate given that this research represents the first attempt to develop and evaluate such scales among Lebanese university students. Although Confirmatory Factor Analysis (CFA) is typically used to further verify the factor structure in an independent sample, conducting CFA was beyond the scope of the present study. Nevertheless, the strong reliability and validity indicators obtained provide solid preliminary evidence supporting the scales' psychometric properties. Future studies with larger or independent samples are encouraged to perform CFA in order to confirm and refine the identified structure. Finally, for reliability measures, this study only focused on internal consistency because the anonymous design of the questionnaire made test-retest reliability unfeasible. While this limits our ability to assess temporal stability, the anonymous approach was prioritized to help students feel safe sharing honest answers about sensitive topics like substance consumption.

## Conclusion

In conclusion, this study was able to develop and validate two robust scales designed to assess the knowledge and attitudes toward psychoactive substance consumption among Lebanese university students. The rigorous psychometric evaluation demonstrated great reliability and validity of both scales, confirming their effectiveness as essential tools for assessing knowledge and attitudes in this demographic. While the scales demonstrated good reliability and construct validity in the current sample, these findings represent an initial psychometric evaluation, and further confirmatory studies are warranted to strengthen their robustness across independent populations. By providing a standardized method for assessing knowledge and attitudes, we improve researchers' ability to explore the factors influencing substance consumption behaviors among university students. Therefore, we anticipate that these scales will serve as valuable sources for future epidemiological studies in Lebanon and potentially in similar contexts.

## Data Availability

The raw data supporting the conclusions of this article will be made available by the authors, without undue reservation.
