# Supplementary material for: From knowledge to attitude: design and initial validation of scales for assessing psychoactive substance consumption among university students
Source: Front Public Health. 2025 Dec 16;13:1713133. doi: 10.3389/fpubh.2025.1713133 (PMC12747911; doi:10.3389/fpubh.2025.1713133)
Supplement: Supplementary file 1 [file Table_5.pdf]

Supplementary Material

Table S5: Comprehensive Bivariate Analysis of Categorical Variables with Knowledge and Attitude Scores

		Knowledge (n (%))			Attitude (n (%))		
		Poor knowledge	Good knowledge	*p*-value	Negative attitude	Positive attitude	*p*-value
**Gender**	Male	128 (73.1)	47 (26.9)	0.791	127 (72.6)	48 (27.4)	0.748
	Female	172 (72.0)	67 (28.0)		170 (71.1)	69 (28.9)	
**Marital status**	Single	289 (72.8)	108 (27.2)	0.629	287 (72.3)	110 (27.7)	0.085
	Married	9 (64.3)	5 (35.7)		7 (50.0)	7 (50.0)	
	Widowed/Divorced	2 (66.7)	1 (33.3)		3 (100.0)	0 (0.0)	
**Province**	Beirut	56 (70.0)	24 (30.0)	0.924	57 (71.3)	23 (28.7)	0.455
	Mount Lebanon	85 (72.6)	32 (27.4)		88 (75.2)	29 (24.8)	
	North	53 (74.6)	18 (25.4)		53 (74.6)	18 (25.4)	
	South	53 (74.6)	18 (25.4)		49 (69.0)	22 (31.0)	
	Nabatiyeh	12 (63.2)	7 (36.8)		10 (52.6)	9 (47.4)	
	Bekaa	36 (72.0)	14 (28.0)		36 (72.0)	14 (28.0)	
**University location**	Beirut	185 (70.9)	76 (29.1)	0.605	185 (70.9)	76 (29.1)	0.909
	Mount Lebanon	88 (76.5)	27 (23.5)		84 (73.0)	31 (27)	
	North	12 (80.0)	3 (20.0)		11 (73.3)	4 (26.7)	
	South	4 (57.1)	3 (42.9)		4 (57.1)	3 (24.9)	
	Nabatiyeh	1 (50.0)	1 (50.0)		2 (100.0)	0 (0.0)	
	Bekaa	10 (71.4)	4 (28.6)		11 (78.6)	3 (21.4)	
**Major**	Medicine	36 (61.0)	23 (39.0)	0.150	40 (67.8)	19 (32.2)	0.949
	Pharmacy	97 (73.5)	35 (26.5)		97 (73.5)	35 (26.5)	
	Nursing	4 (57.1)	3 (42.9)		4 (57.1)	3 (42.9)	
	Arts & Sciences	89 (74.2)	31 (25.8)		88 (73.3)	32 (26.7)	
	Business & Management	26 (70.3)	11 (29.7)		27 (73.0)	10 (27.0)	
	Arts & Design	24 (82.8)	5 (17.2)		20 (69.0)	9 (31.0)	
	Engineering	24 (82.8)	5 (17.2)		20 (69.0)	9 (31.0)	
	Other	0 (0.0)	1 (100.0)		1 (100.0)	0 (0.0)	
**Year of study**	First year	67 (81.7)	15 (18.3)	0.108	59 (72.0)	23 (28.0)	0.992
	Second year	72 (69.2)	32 (30.8)		75 (72.1)	29 (27.9)	
	≥ 3rd year	161 (70.6)	67 (29.4)		163 (71.5)	65 (28.5)	
**Students' grades**	Good grade (A or B)	204 (70.6)	85 (29.4)	**0.030**	207 (71.6)	82 (28.4)	0.770

	Passing grade (C)		79 (73.1)	29 (26.9)		79 (73.1)	29 (26.9)	
	Failing grade (D or F)		17 (100.0)	0 (0.0)		11 (64.7)	6 (35.3)	
**Place of residence**	On-campus alone		5 (45.5)	6 (54.5)	0.362	4 (36.4)	7 (63.6)	0.069
	On-campus with roommate		29 (76.3)	9 (23.7)		30 (78.9)	8 (21.1)	
	Off-campus alone		26 (74.3)	9 (25.7)		26 (74.3)	9 (25.7)	
	Off-campus with roommate		3 (75.0)	1 (25.0)		2 (50.0)	2 (50.0)	
	Off-campus with family		237 (72.7)	89 (27.3)		235 (72.1)	91 (27.9)	
**Work status**	Student only		201 (74.4)	69 (25.6)	0.219	188 (69.6)	82 (30.4)	0.546
	Employed part-time		59 (72.8)	22 (27.2)		63 (77.8)	18 (22.2)	
	Employed full-time		34 (66.7)	17 (33.3)		37 (72.5)	14 (27.5)	
	Trainee/intern		6 (50.0)	6 (50.0)		9 (75.0)	3 (25.0)	
**Parent's marital status**	Living together		247 (71.0)	101 (29.0)	0.120	247 (71.0)	101 (29.0)	0.429
	Not together		53 (80.3)	13 (19.7)		50 (75.8)	16 (24.2)	
**Parent's education**	Mother	Postgraduate	43 (66.2)	22 (33.8)	0.419	43 (66.2)	22 (33.8)	0.454
		Graduate	127 (72.6)	48 (27.4)		130 (74.3)	45 (25.7)	
		Undergraduate	130 (74.7)	44 (25.3)		124 (71.3)	50 (28.7)	
	Father	Postgraduate	46 (69.7)	20 (30.3)	0.851	51 (77.3)	15 (22.7)	0.500
		Graduate	113 (73.4)	41 (26.6)		107 (69.5)	47 (30.5)	
		Undergraduate	141 (72.7)	53 (27.3)		139 (71.6)	55 (28.4)	
**CFPB**	Very low		1 (50.0)	1 (50.0)	0.241	2 (100.0)	0 (0.0)	0.681
	Low		14 (87.5)	2 (12.5)		14 (87.5)	2 (12.5)	
	Medium-low		73 (65.2)	39 (34.8)		79 (70.5)	33 (29.5)	
	Medium-high		138 (75.0)	46 (25.0)		132 (71.7)	52 (28.3)	
	High		63 (75.0)	21 (25.0)		60 (71.4)	24 (28.6)	
	Very high		11 (68.8)	5 (31.2)		10 (62.5)	6 (37.5)	
**Exercise**	No		126 (71.2)	51 (28.8)	0.615	132 (74.6)	45 (25.4)	0.268
	Yes		174 (73.4)	63 (26.6)		165 (69.6)	72 (30.4)	
**Presence of mental illness**	No		289 (72.4)	110 (27.6)	0.939	286 (71.7)	113 (28.3)	0.889
	Yes		11 (73.3)	4 (26.7)		11 (73.3)	4 (26.7)	
**Family member has mental illness**	No		277 (71.9)	108 (28.1)	0.392	273 (70.9)	112 (29.1)	0.172
	Yes		23 (79.3)	6 (20.7)		24 (82.8)	5 (17.2)	
**Family member has alcoholism**	No		293 (72.0)	114 (28.0)	0.197	291 (71.5)	116 (28.5)	0.679
	Yes		7 (100.0)	0 (0.0)		6 (85.7)	1 (14.3)	
**Family member has drug abuse problem**	No		294 (72.1)	114 (27.9)	0.194	291 (71.3)	117 (28.7)	0.191
	Yes		6 (100.0)	0 (0.0)		6 (100.0)	0 (0.0)	

**Family member has heavy smoking problem**	No	230 (72.8)	86 (27.2)	0.793	233 (73.7)	83 (26.3)	0.105
	Yes	70 (71.4)	28 (28.6)		64 (65.3)	34 (34.7)	
**GAD-7 ranges**	None to minimal anxiety	57 (72.2)	22 (27.8)	0.911	59 (74.7)	20 (25.3)	0.936
	Mild anxiety	98 (70.5)	41 (29.5)		99 (71.2)	40 (28.8)	
	Moderate anxiety	63 (73.3)	23 (26.7)		61 (70.9)	25 (29.1)	
	Severe anxiety	82 (74.5)	28 (25.5)		78 (70.9)	32 (29.1)	
**PHQ-9 ranges**	Minimal depression	56 (70.9)	23 (29.1)	0.645	60 (75.9)	19 (24.1)	0.649
	Mild depression	98 (69.5)	43 (30.5)		101 (71.6)	40 (28.4)	
	Moderate depression	58 (71.6)	23 (28.4)		57 (70.4)	24 (29.6)	
	Moderately severe depression	46 (76.7)	14 (23.3)		39 (65.0)	21 (35.0)	
	Severe depression	42 (79.2)	11 (20.8)		40 (75.5)	13 (24.5)	
**Received information about substance abuse**	No	105 (78.4)	29 (21.6)	0.063	101 (75.4)	33 (24.6)	0.256
	Yes	195 (69.6)	85 (30.4)		196 (70.0)	84 (30.0)	
**Students aware of nasal inhalation (e.g., snorting, nasal spray, etc.)**	No	51 (83.6)	10 (16.4)	**0.035**	43 (70.5)	18 (29.5)	0.815
	Yes	249 (70.5)	104 (29.5)		254 (72.0)	99 (28.0)	
**Students aware of pulmonary inhalation (e.g., cigarette smoking)**	No	44 (71.0)	18 (29.0)	0.785	45 (72.6)	17 (27.4)	0.863
	Yes	255 (72.6)	96 (27.4)		251 (71.5)	100 (28.5)	
**Students aware of transdermal route (e.g., patch)**	No	146 (74.1)	51 (25.9)	0.474	138 (70.1)	59 (29.9)	0.467
	Yes	154 (71.0)	63 (29.0)		159 (73.3)	58 (26.7)	
**Students aware of injection route (e.g., IV, IM, SQ, etc.)**	No	51 (76.1)	16 (23.9)	0.464	52 (77.6)	15 (22.4)	0.244
	Yes	249 (71.8)	98 (28.2)		245 (70.6)	102 (29.4)	
**Students aware of oral route: tablets**	No	86 (76.1)	27 (23.9)	0.309	82 (72.6)	31 (27.4)	0.819
	Yes	214 (71.1)	87 (28.9)		215 (71.4)	86 (28.6)	
**Students aware of oral route: chewing gum**	No	132 (75.9)	42 (24.1)	0.188	122 (70.1)	52 (29.9)	0.532
	Yes	168 (70.0)	72 (30.0)		175 (72.9)	65 (27.1)	
**Attitude**	Negative	210 (70.7)	87 (29.3)	0.202			
	Positive	90 (76.9)	27 (23.1)				
