# Supplementary material for: From knowledge to attitude: design and initial validation of scales for assessing psychoactive substance consumption among university students
Source: Front Public Health. 2025 Dec 16;13:1713133. doi: 10.3389/fpubh.2025.1713133 (PMC12747911; doi:10.3389/fpubh.2025.1713133)
Supplement: Supplementary file 2 [file Data_Sheet_1.pdf]

Questionnaire

The objective of this questionnaire is to gather information in order to conduct a scientific research study related to the use of psychotropic substances among Lebanese university students. The use of alcohol, tobacco, illicit substances or medications is referred to as Psychotropic Substance Consumption (PSC) in this document.

Rest assured that the information you provide will be treated with high confidentiality and will only be used for scientific purposes. Please answer the questions honestly and accurately.
Thank you in advance.

Please check **all** the below in order to proceed to the questionnaire:

☐ I have read and understood the above information

☐ I am currently a student attending a Lebanese university

☐ I agree to participate in this study

☐ I understand that my participation is voluntary

☐ I understand that my data will be kept confidential

I- SOCIO-DEMOGRAPHIC CHARACTERISTICS:

1. Age: _____________ years
2. Gender: ☐ Male ☐ Female
3. What is your current marital status?

   ☐ Married ☐ Divorced ☐ Widowed ☐ Single
4. Where are you originally from?

   ☐ Beirut ☐ Mount Lebanon ☐ North ☐ South

   ☐ Akkar ☐ Baalbeck-Hermel ☐ Nabatiyeh ☐ Bekaa

   ☐ Not from Lebanon (specify): ____
5. University: ______________________________ Location: ______________________________
6. Major:

   ____
7. Current year of study (2023-2024): ☐ First ☐ Second ☐ Third ☐ Fourth ☐ Other: ____
8. What is your current Grade Point Average (GPA) or yearly average? ________________________

   ☐ I don’t have a GPA yet
9. Where do you live?

   ☐ On-campus alone ☐ On-campus with roommate

   ☐ Off-campus alone ☐ Off-campus with family

10. How many individuals are living in your family home (including yourself)? _________________

11. What is your household income per month (by all your family) (L.L.)?

☐ < 10.000.000 ☐ <10.000.000 – 20.000.000 ☐ <20.000.000 – 30.000.000

☐<30.000.000 –40.000.000 ☐<40.000.000 – 50.000.000 ☐ > 50.000.000

12. What is your current work status?

☐ Unemployed ☐ Employed part-time and student ☐ Employed part-time and student

☐ Trainee_intern ☐ Student ☐ Other, specify: _____________________

13. Marital status of your parents:

☐ Mother and father living together ☐ Separated ☐ Divorced

☐ Widowed ☐ Other, specify: ______________________

14. Your parents' highest level of education:

**Mother**	**Father**
☐ Doctorate	☐ Doctorate
☐ Masters	☐ Masters
☐ Bachelors	☐ Bachelors
☐ School	☐ School
☐ Did not graduate from school	☐ Did not graduate from school

15. How do you classify your financial status?

**Before 2019 (before economic crisis/Covid)**	**2024 (Now)**
☐ Comfortable	☐ Comfortable
☐ It's fine	☐ It's fine
☐ Just as needed	☐ Just as needed
☐ Have to be careful	☐ Have to be careful
☐ Tough	☐ Tough
☐ Can't make it without loans	☐ Can't make it without loans

16. Financial Wellbeing Scale:

Part 1: How well does this statement describe you or your situation?

**This statement describes me**	**Completely**	**Very well**	**Somewhat**	**Very little**	**Not at all**
1. I could handle a major unexpected expense	☐	☐	☐	☐	☐

2. I am securing my financial future	☐	☐	☐	☐	☐
3. Because of my money situation, I feel like I will never have the things I want in life	☐	☐	☐	☐	☐
4. I can enjoy life because of the way I'm managing my money	☐	☐	☐	☐	☐
5. I am just getting by financially	☐	☐	☐	☐	☐
6. I am concerned that the money I have or will save won't last	☐	☐	☐	☐	☐

Part 2: How often does this statement apply to you?

**This statement applies to me**	**Always**	**Often**	**Sometimes**	**Rarely**	**Never**
1. Giving a gift for a wedding, birthday or other occasion would put a strain on my finances for the month	☐	☐	☐	☐	☐
2. I have money left over at the end of the month	☐	☐	☐	☐	☐
3. I am behind with my finances	☐	☐	☐	☐	☐
4. My finances control my life	☐	☐	☐	☐	☐

II- HEALTH CHARACTERISTICS:

1. Do you exercise or play any sport?

☐ No ☐ Yes (specify which sport): ____________________________

☐ If yes, how often do you exercise?

☐ Once a week ☐ twice a week ☐ 3 or more times a week

2. Please list all known diseases that you currently have:

____

____

☐ No current disease

3. Please list all surgical procedures of the past:

____

☐ No major surgical procedures

4. Which medication(s) do you currently use (indication):

____

____

5. Does any of your family members (parents, brothers/sisters) have the following problems?

☐ Mental illness ☐ Drug abuse

☐ Alcoholism ☐ Heavy smoking (please specify): ___________________

☐ Other illnesses (please specify): ____

☐ No, they don't have any illnesses

EMOTIONAL HEALTH - STRESS LEVEL:

A. Generalized Anxiety Disorder 7-item (GAD-7) scale

**Over the last 2 weeks, how often have you been bothered by the following problems?**	Not at all sure	Several days	Over half the days	Nearly every day
1. Feeling nervous, anxious, or on edge	☐	☐	☐	☐
2. Not being able to stop or control worrying	☐	☐	☐	☐
3. Worrying too much about different things	☐	☐	☐	☐
4. Trouble relaxing	☐	☐	☐	☐
5. Being so restless that it's hard to sit still	☐	☐	☐	☐
6. Becoming easily annoyed or irritable	☐	☐	☐	☐
7. Feeling afraid as if something awful might happen	☐	☐	☐	☐

If you checked off any problems, how difficult have these made it for you to do your work, take care of things at home, or get along with other people?

☐ Not difficult at all ☐ Somewhat difficult

☐ Very difficult ☐ Extremely difficult

B. Patient Health Questionnaire – 9 (PHQ9) Scale :

How often have you been bothered by the following over the past 2 weeks?

Factor	Not at all	Several days	More than half the days	Nearly every day
1. Little interest or pleasure in doing things?	☐	☐	☐	☐
2. Feeling down, depressed, or hopeless?	☐	☐	☐	☐
3. Trouble falling or staying asleep, or sleeping too much?	☐	☐	☐	☐
4. Feeling tired or having little energy?	☐	☐	☐	☐
5. Poor appetite or overeating?	☐	☐	☐	☐
6. Feeling bad about yourself — or that you are a failure or have let yourself or your family down?	☐	☐	☐	☐
7. Trouble concentrating on things, such as reading the newspaper or watching television?	☐	☐	☐	☐
8. Moving or speaking so slowly that other people could have noticed? Or so fidgety or restless that you have been moving a lot more than usual?	☐	☐	☐	☐
9. Thoughts that you would be better off dead, or thoughts of hurting yourself in some way?	☐	☐	☐	☐

If you checked off any of the problems listed above, how difficult have those problems made it for you to do your work, take care of things at home, or get along with other people?

☐ Not difficult at all ☐ Somewhat difficult

☐ Very difficult ☐ Extremely difficult

III- KNOWLEDGE, ATTITUDES AND PRACTICES (KAP) INFORMATION:

N.B: The use of alcohol, tobacco or drugs is referred to as Psychotropic Substance Consumption (PCS) in this document

A-Knowledge towards psychotropic substance use (reflects the knowledge about psychotropic substances)

1. Have you ever received information about drug abuse? ☐ No ☐ Yes ☐ I don't remember

2. If yes, from where have you received information? (Multiple answers possible)

Information	No	Yes
TV/Movie	☐	☐
Internet	☐	☐
School	☐	☐
Family	☐	☐
Friends	☐	☐
N/A	☐	☐

Questions	Strongly agree	Agree	Neutral	Disagree	Strongly disagree
1. The rate of drug abuse has increased over the years	☐	☐	☐	☐	☐
2. Psychotropic substance consumption affects how the brain works	☐	☐	☐	☐	☐
3. Psychotropic substances may cause changes in mood, awareness, thoughts, feelings, or behavior	☐	☐	☐	☐	☐
4. When people engage in substance use, they get a lift and feel of instant happiness and pleasure	☐	☐	☐	☐	☐
5. Peer pressure influences substance abuse	☐	☐	☐	☐	☐
6. Family background is the leading cause of substance abuse	☐	☐	☐	☐	☐
7. Students who abuse drugs have low self-esteem	☐	☐	☐	☐	☐
8. Women have low tolerance to alcohol than men	☐	☐	☐	☐	☐
9. High consumption of marijuana decreases sexual hormones	☐	☐	☐	☐	☐
10. Health problems can result from taking psychotropic substances (hypertension, liver and kidney damage, etc.	☐	☐	☐	☐	☐
11. Psychotropic abuse makes you more vulnerable to HIV and unwanted pregnancy	☐	☐	☐	☐	☐

Which of the following methods/routes of taking drugs are you aware of?

Methods/routes	No	Yes
**Transdermal**: patch	☐	☐
**Pulmonary inhalation**: cigarette smoking	☐	☐
**Nasal inhalation**: snorting, nasal spray, etc.	☐	☐
**Injection**: intravenous, intramuscular, subcutaneous, etc.	☐	☐
**Oral**: tablet	☐	☐
**Oral**: chewing gum	☐	☐

B-Attitude towards psychotropic substance use (defined as the university students' opinions and beliefs towards awareness and socio-cultural perspectives)

Questions	Strongly agree	Agree	Neutral	Disagree	Strongly disagree
1. Do you believe that most students who abuse drugs come from poor backgrounds?	☐	☐	☐	☐	☐
2. Do you think that students who abuse drugs are often violent?	☐	☐	☐	☐	☐
3. Substance abuse is a crime and should be punishable by law?	☐	☐	☐	☐	☐
4. Do you feel comfortable taking psychotropic substances without physician's prescription?	☐	☐	☐	☐	☐
5. Taking psychotropic medication without prescription from time to time is not harmful	☐	☐	☐	☐	☐
6. Do you think you have all the information you need about psychotropic substances?	☐	☐	☐	☐	☐
7. The benefit of taking these drugs outweigh their risks	☐	☐	☐	☐	☐

8. Are these substances readily available to you?	☐	☐	☐	☐	☐
9. Do you feel pressured to take any kind of substance by your friends?	☐	☐	☐	☐	☐
10. Do you intend to use any substance in the next 6 months?	☐	☐	☐	☐	☐
If you are with some friends at a party, and one of them offered you drugs, would you:	☐ Take some	☐ Think about it	☐ Definitely say no		

C-Practices towards psychotropic substance use (corresponds to the students' use and abuse of psychotropic substances) ----- **The ASSIST TOOL**

Question 1

**In your life, which of the following substances have you ever used?** **(NON-MEDICAL USE ONLY)**	**No**	**Yes**
a. Tobacco products (cigarettes, chewing tobacco, cigars, etc)	☐	☐
b. Alcoholic beverages (beer, wine, spirits, etc.)	☐	☐
c. Cannabis (marijuana, pot, grass, hash, etc.)	☐	☐
d. Cocaine (coke, crack, etc.)	☐	☐
e. Amphetamine type stimulants (speed, meth, ecstasy, etc.)	☐	☐
f. Inhalants (nitrous, glue, paint thinner, etc.)	☐	☐
g. Sedatives or sleeping pills (Diazepam, Valium, Alprazolam, Xanax, lorazepam, Midazolam, Flunitrazepam, tranquilizers, etc.)	☐	☐
h. Hallucinogens (LSD, acid, mushrooms, Ketamine, PCP, Special K, ecstasy, etc.)	☐	☐
i. Opioids (tramadol, codeine, heroin, opium, morphine, methadone, buprenorphine, Detromethorphan,etc.)	☐	☐
j. Gabapentinoids (Gabapentin (Neurontin), Pregabalin, (Lyrica) etc.)	☐	☐
k. Any other drugs. Please specify:	☐	☐

If you answer "no" to all questions, or do not provide any answers, you are done.
If you answer "yes" to any question, then proceed to Question 2

Question 2

In the <u>past three months</u>, how often have you used the substances you mentioned?	Never	Once or twice	Monthly	Weekly	Daily or almost daily
a. Tobacco products (cigarettes, chewing tobacco, cigars, etc)	☐	☐	☐	☐	☐
b. Alcoholic beverages (beer, wine, spirits, etc.)	☐	☐	☐	☐	☐
c. Cannabis (marijuana, pot, grass, hash, etc.)	☐	☐	☐	☐	☐
d. Cocaine (coke, crack, etc.)	☐	☐	☐	☐	☐
e. Amphetamine type stimulants (speed, meth, ecstasy, etc.)	☐	☐	☐	☐	☐
f. Inhalants (nitrous, glue, paint thinner, etc.)	☐	☐	☐	☐	☐
g. Sedatives or sleeping pills (Diazepam, Valium, Alprazolam, Xanax, lorazepam, Midazolam, Flunitrazepam, tranquilizers, etc.)	☐	☐	☐	☐	☐
h. Hallucinogens (LSD, acid, mushrooms, Ketamine, PCP, Special K, ecstasy, etc.)	☐	☐	☐	☐	☐
i. Opioids (tramadol, codeine, heroin, opium, morphine, methadone, buprenorphine, Detromethorphan,etc.)	☐	☐	☐	☐	☐
j. Gabapentinoids (Gabapentin (Neurontin), Pregabalin, (Lyrica) etc.)	☐	☐	☐	☐	☐
k. Any other drug. Please specify:	☐	☐	☐	☐	☐

If you answer "never" for all drugs on question 2, or do not provide any answers, skip toQuestion 6. Otherwise proceed to Question 3.

Question 3

During the <u>past three months</u>, how often have you had a strongdesire or urge to use?	Never	Once or twice	Monthly	Weekly	Daily or almost daily
a. Tobacco products (cigarettes, chewing tobacco, cigars, etc)	☐	☐	☐	☐	☐
b. Alcoholic beverages (beer, wine, spirits, etc.)	☐	☐	☐	☐	☐
c. Cannabis (marijuana, pot, grass, hash, etc.)	☐	☐	☐	☐	☐
d. Cocaine (coke, crack, etc.)	☐	☐	☐	☐	☐
e. Amphetamine type stimulants (speed, meth, ecstasy, etc.)	☐	☐	☐	☐	☐
f. Inhalants (nitrous, glue, paint thinner, etc.)	☐	☐	☐	☐	☐

g. Sedatives or sleeping pills (Diazepam, Valium, Alprazolam, Xanax, lorazepam, Midazolam, Flunitrazepam, tranquilizers, etc.)	☐	☐	☐	☐	☐
h. Hallucinogens (LSD, acid, mushrooms, Ketamine, PCP, Special K, ecstasy, etc.)	☐	☐	☐	☐	☐
i. Opioids (tramadol, codeine, heroin, opium, morphine, methadone, buprenorphine, Detromethorphan,etc.)	☐	☐	☐	☐	☐
j. Gabapentinoids (Gabapentin (Neurontin), Pregabalin, (Lyrica) etc.)	☐	☐	☐	☐	☐
k. Any other drug. Please specify:	☐	☐	☐	☐	☐

Question 4

**During the <u>past three months</u>, how often has your use led to health, social, legal or financial problems?**	**Never**	**Once or twice**	**Monthly**	**Weekly**	**Daily or almost daily**
a. Tobacco products (cigarettes, chewing tobacco, cigars, etc)	☐	☐	☐	☐	☐
b. Alcoholic beverages (beer, wine, spirits, etc.)	☐	☐	☐	☐	☐
c. Cannabis (marijuana, pot, grass, hash, etc.)	☐	☐	☐	☐	☐
d. Cocaine (coke, crack, etc.)	☐	☐	☐	☐	☐
e. Amphetamine type stimulants (speed, meth, ecstasy, etc.)	☐	☐	☐	☐	☐
f. Inhalants (nitrous, glue, paint thinner, etc.)	☐	☐	☐	☐	☐
g. Sedatives or sleeping pills (Diazepam, Valium, Alprazolam, Xanax, lorazepam, Midazolam, Flunitrazepam, tranquilizers, etc.)	☐	☐	☐	☐	☐
h. Hallucinogens (LSD, acid, mushrooms, Ketamine, PCP, Special K, ecstasy, etc.)	☐	☐	☐	☐	☐
i. Opioids (tramadol, codeine, heroin, opium, morphine, methadone, buprenorphine, Detromethorphan,etc.)	☐	☐	☐	☐	☐
j. Gabapentinoids (Gabapentin (Neurontin), Pregabalin, (Lyrica) etc.)	☐	☐	☐	☐	☐
k. Any other drug. Please specify:	☐	☐	☐	☐	☐

Question 5

During the <u>past three months</u>, how often have you failed todo what was normally expected of you because of your use of	Never	Once or twice	Monthly	Weekly	Daily or almost daily
a. Tobacco products (cigarettes, chewing tobacco, cigars, etc)	☐	☐	☐	☐	☐
b. Alcoholic beverages (beer, wine, spirits, etc.)	☐	☐	☐	☐	☐
c. Cannabis (marijuana, pot, grass, hash, etc.)	☐	☐	☐	☐	☐
d. Cocaine (coke, crack, etc.)	☐	☐	☐	☐	☐
e. Amphetamine type stimulants (speed, meth, ecstasy, etc.)	☐	☐	☐	☐	☐
f. Inhalants (nitrous, glue, paint thinner, etc.)	☐	☐	☐	☐	☐
g. Sedatives or sleeping pills (Diazepam, Valium, Alprazolam, Xanax, lorazepam, Midazolam, Flunitrazepam, tranquilizers, etc.)	☐	☐	☐	☐	☐
h. Hallucinogens (LSD, acid, mushrooms, Ketamine, PCP, Special K, ecstasy, etc.)	☐	☐	☐	☐	☐
i. Opioids (tramadol, codeine, heroin, opium, morphine, methadone, buprenorphine, Detromethorphan,etc.)	☐	☐	☐	☐	☐
j. Gabapentinoids (Gabapentin (Neurontin), Pregabalin, (Lyrica) etc.)	☐	☐	☐	☐	☐
k. Any other drug. Please specify:	☐	☐	☐	☐	☐

Question 6

Has a friend or relative or anyone else ever expressed concernabout your use of	No, never	Yes, in the past 3 months	Yes, but not in the past 3 months
a. Tobacco products (cigarettes, chewing tobacco, cigars, etc)	☐	☐	☐
b. Alcoholic beverages (beer, wine, spirits, etc.)	☐	☐	☐
c. Cannabis (marijuana, pot, grass, hash, etc.)	☐	☐	☐
d. Cocaine (coke, crack, etc.)	☐	☐	☐
e. Amphetamine type stimulants (speed, meth, ecstasy, etc.)	☐	☐	☐
f. Inhalants (nitrous, glue, paint thinner, etc.)	☐	☐	☐

g. Sedatives or sleeping pills (Diazepam, Valium, Alprazolam, Xanax, lorazepam, Midazolam, Flunitrazepam, tranquilizers, etc.)	☐	☐	☐
h. Hallucinogens (LSD, acid, mushrooms, Ketamine, PCP, Special K, ecstasy, etc.)	☐	☐	☐
i. Opioids (tramadol, codeine, heroin, opium, morphine, methadone, buprenorphine, Detromethorphan,etc.)	☐	☐	☐
j. Gabapentinoids (Gabapentin (Neurontin), Pregabalin, (Lyrica) etc.)	☐	☐	☐
k. Any other drug. Please specify:	☐	☐	☐

Question 7

**Have you ever tried and failed to control, cut down or stop using?**	**No, never**	**Yes, in the past 3 months**	**Yes, but not in the past 3 months**
a. Tobacco products (cigarettes, chewing tobacco, cigars, etc)	☐	☐	☐
b. Alcoholic beverages (beer, wine, spirits, etc.)	☐	☐	☐
c. Cannabis (marijuana, pot, grass, hash, etc.)	☐	☐	☐
d. Cocaine (coke, crack, etc.)	☐	☐	☐
e. Amphetamine type stimulants (speed, meth, ecstasy, etc.)	☐	☐	☐
f. Inhalants (nitrous, glue, paint thinner, etc.)	☐	☐	☐
g. Sedatives or sleeping pills (Diazepam, Valium, Alprazolam, Xanax, lorazepam, Midazolam, Flunitrazepam, tranquilizers, etc.)	☐	☐	☐
h. Hallucinogens (LSD, acid, mushrooms, Ketamine, PCP, Special K, ecstasy, etc.)	☐	☐	☐
i. Opioids (tramadol, codeine, heroin, opium, morphine, methadone, buprenorphine, Detromethorphan,etc.)	☐	☐	☐
j. Gabapentinoids (Gabapentin (Neurontin), Pregabalin, (Lyrica) etc.)	☐	☐	☐
k. Any other drug. Please specify:	☐	☐	☐

Question 8

**Have you ever used any drug by injection? (NON-MEDICAL USE ONLY)**	**No, never**	**Yes, in thepast 3 months**	**Yes, but not in the past 3 months**
	☐	☐	☐

If you answer "Yes, in the past 3 months" for Question 8, you should answer the two questions below

Extra drug injection questions

During the past three months, how often have you injected drugs?	Once per week or less ☐	More than once per week ☐
During the past three months, have you ever injected drugs three or more days in a row?	Yes ☐	No ☐

Question 9

What are your motives and circumstances for psychoactive substance consumption? (Multiple answers possible)

Motive	No	Yes
For pleasure / recreational	☐	☐
For sedation / to induce sleep	☐	☐
To relieve anxiety and stress	☐	☐
To improve concentration / study difficulties	☐	☐
To relieve depression	☐	☐
Because I am lonely	☐	☐

☐ Other, please specify: ______________________________
